# Supplementary material for: High-Pressure Inactivation of Bacillus cereus in Human Breast Milk
Source: Foods. 2023 Nov 24;12(23):4245. doi: 10.3390/foods12234245 (PMC10706202; doi:10.3390/foods12234245)
Supplement: Supplementary file 1 [file foods-12-04245-s001.zip › Supplementary data_Tables.pdf]

S1. Results of microbiological analysis performed in samples inoculated at Department of Biochemistry and Microbiology, University of Chemistry and Technology Prague.

**Experiment No.**	**Sample ID**	**Total amount intended for inoculation**		**Really inoculated number of *B. cereus* (verified from randomly selected samples)**		**Pressurization methods**	
		**Total of CFU/mL**	**Number of spores**	**Inoculated really**	**Number of spores**	**Method P1 - treatment in cycles**	**Method P2 - continuous treatment**
	1063 A1			9.10 x 10^{6}	3.50 x 10^{6}	1.90 x 10^{2}	
	1063 A2			1.10 x 10^{7}	4.10 x 10^{6}		2.00 x 10^{3}
	1063 B1					2.90 x 10^{2}	
	1063 B2						2.90 x 10^{3}
	1064 A1					1.10 x 10^{2}	
	1064 A2						1.80 x 10^{3}
	1064 B1					1.30 x 10^{2}	
	1064 B2						1.20 x 10^{3}
	1065 A1					2.70 x 10^{2}	
**I**	1065 A2	1.10 x 10^{9}	4.10 x 10^{8}				1.80 x 10^{3}
	1065 B1					1.20 x 10^{2}	
	1065 B2						1.40 x 10^{3}
	1066 A1					1.00 x 10^{2}	
	1066 A2						2.20 x 10^{3}
	1066 B1			7.90 x 10^{6}	3.80 x 10^{6}	2.80 x 10^{2}	
	1066 B2			7.80 x 10^{6}	4.00 x 10^{6}		1.60 x 10^{3}
	1067 A1					1.30 x 10^{2}	
	1067 A2						1.60 x 10^{3}
	1067 B1					2.90 x 10^{2}	
	1067 B2						2.30 x 10^{3}

	BLANK 1			8.90×10^{6}	3.90×10^{6}	1.80×10^{7}	1.70×10^{7}
	BLANK 2			9.10×10^{6}	3.80×10^{6}	3.00×10^{5}	3.00×10^{5}
	1068 B1					53	
	1068 B2						6.40×10^{2}
	1068 C1			5.00×10^{6}	1.70×10^{5}	75	
	1068 C2			7.90×10^{6}	1.60×10^{5}		5.30×10^{2}
	1070 A1					90	
	1070 A2						4.80×10^{2}
	1860 A1					1.10×10^{2}	
	1860 A2						4.00×10^{2}
	1860 B1					1.30×10^{2}	
	1860 B2						5.50×10^{2}
	1861 A1			3.70×10^{6}	1.30×10^{5}	1.50×10^{2}	
**II**	1861 A2	5.30×10^{8}	1.90×10^{7}	2.90×10^{6}	1.20×10^{5}		5.60×10^{2}
	1861 B1					1.40×10^{2}	
	1861 B2						4.30×10^{2}
	1869 A1					1.30×10^{2}	
	1869 A2						6.50×10^{2}
	1690 A1					1.40×10^{2}	
	1690 A2						5.80×10^{2}
	1690 B1					83	
	1690 B2						5.30×10^{2}
	BLANK 1			4.10×10^{6}	1.30×10^{5}	6.40×10^{6}	7.10×10^{6}
	BLANK 2			3.70×10^{6}	1.50×10^{5}	1.00×10^{5}	1.40×10^{5}
III	1899 A1	4.30×10^{7}	1.70×10^{4}			< 5	< 5
	1899 A2					< 5	< 5

1899 B1	3.30 x 10^5	1.25 x 10^2	< 5	< 5
1899 B2	3.10 x 10^5	1.15 x 10^2	< 5	< 5
1908 A1			< 5	< 5
1908 A2			< 5	< 5
1908 B1			< 5	< 5
1908 B2			< 5	< 5
1979 A1			< 5	< 5
1979 A2			< 5	< 5
1979 B1			< 5	< 5
1979 B2			< 5	< 5
2038 A1			< 5	< 5
2038 A2			< 5	< 5
2038 B1			< 5	< 5
2038 B2			< 5	< 5
2157 A1	2.90 x 10^5	95	< 5	< 5
2157 A2	2.80 x 10^5	1.28 x 10^2	< 5	< 5
2157 B1			< 5	< 5
2157 B2			< 5	< 5
BLANK 1	2.90 x 10^5	1.35 x 10^2	5.50 x 10^5	7.50 x 10^5
BLANK 2	2.70 x 10^5	1.11 x 10^2	1.10 x 10^2	90

S2. Results of microbiological analysis performed in samples inoculated at Department of Clinical Microbiology, University Hospital Hradec Králové.

Experiment No.	Sample ID	Total amount intended for inoculation	Total number of *B. cereus*		Pressurization methods			
					Method P1 - treatment in cycles		Method P2 - continuous treatment	
			Before inoculation	After inoculation	Quantitative assessment CFU/mL	Qualitative assessment after multiplication in THB	Quantitative assessment CFU/mL	Qualitative assessment after multiplication in THB
VI	M1	37	26	87	0	negative	0	negative
	M2	37	0	67	0	negative	0	negative
	M3	28	2	49	0	negative	0	negative
	M4	28	0	72	0	negative	0	negative
	M6	25	6	62	0	negative	0	negative
	M7	33	2	71	0	negative	0	negative
	M8	33	294	344	0	negative	0	negative
	M10	13	0	73	0	negative	0	negative
	BLANK 1	25	2	65	0	negative	**35**	**positive**
	BLANK 2	13	4	60	**39**	**positive**	0	negative
VII	M 1	87	72	147	0	negative	0	negative
	M 2	87	52	107	0	negative	0	negative
	M 3	87	0	55	0	negative	0	negative
	M 4	87	16	73	0	negative	0	negative
	M 5	79	2	45	0	negative	0	negative
	M 6	79	14	107	0	negative	0	negative
	M 7	79	28	93	0	negative	0	negative
	M 8	79	2	51	0	negative	0	negative
	M 9	72	0	42	0	negative	0	negative
	M 10	72	178	213	0	negative	**3**	**positive**
	BLANK	72	0	31	**304**	**positive**	**$> 10^3$**	**poz.**

	M 1	44	0	72	0	negative	0	negative
	M 2	44	6	70	0	negative	0	**positive**
	M 3	47	18	75	0	**positive**	0	**positive**
	M 4	47	18	71	0	negative	**3**	negative
	M 5	39	148	191	0	negative	0	negative
VIII	M 6	39	0	34	0	negative	0	negative
	M 7	35	48	113	0	negative	0	**positive**
	M 8	35	24	79	1	negative	**3**	negative
	M 9	27	0	45	0	negative	**1**	negative
	M 10	27	0	61	0	negative	0	**positive**
	BLANK	35	0	55	**> 10^3**	**positive**	**> 10^3**	**positive**

S3. Results of residual of *B. cerus* (in % CFU/mL).

**Residual CFU/mL after pressurization of initial values in %**		
**Method**	**P1**	**P2**
	0.01	0.05
	0.01	0.04
	0.04	0.33
	0.12	0.47
	1.21	0.93
	0.03	0.11
**(Mean ± SD) CFU/mL**	0.24 ± 0.48	0.32 ± 0.34
