# Supplementary figures and images for: High-Pressure Inactivation of Bacillus cereus in Human Breast Milk
Source: Foods. 2023 Nov 24;12(23):4245. doi: 10.3390/foods12234245 (PMC10706202; doi:10.3390/foods12234245)

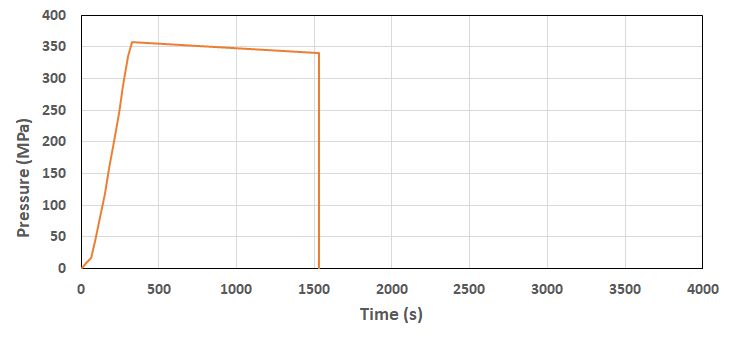

Supplement: Supplementary file 1 [file foods-12-04245-s001.zip › Figure S10_UCT_Experiment II_Continuous pressurization.jpg]

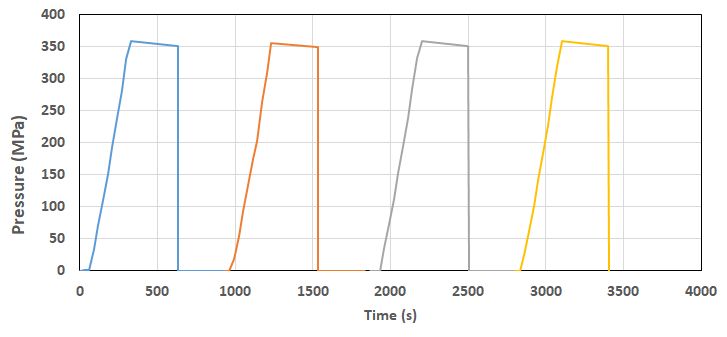

Supplement: Supplementary file 1 [file foods-12-04245-s001.zip › Figure S11_UCT_Experiment III_Pressurization in cycles.jpg]

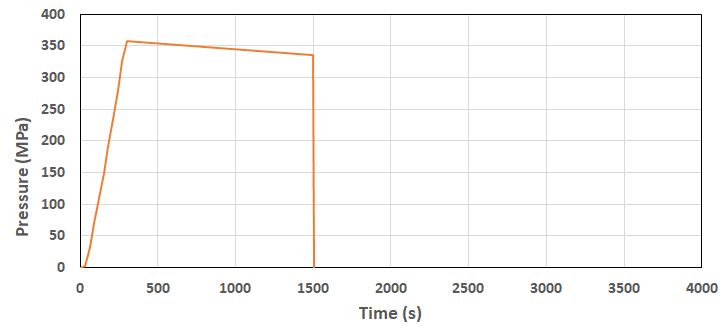

Supplement: Supplementary file 1 [file foods-12-04245-s001.zip › Figure S12_UCT_Experiment III_Continuous pressurization.jpg]

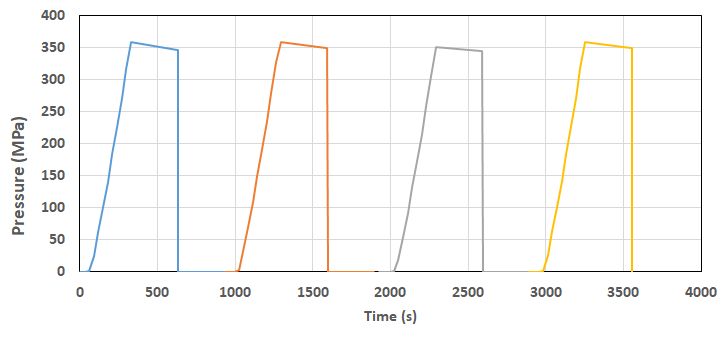

Supplement: Supplementary file 1 [file foods-12-04245-s001.zip › Figure S13_UCT_Experiment_IV_Pressurization in cycles.jpg]

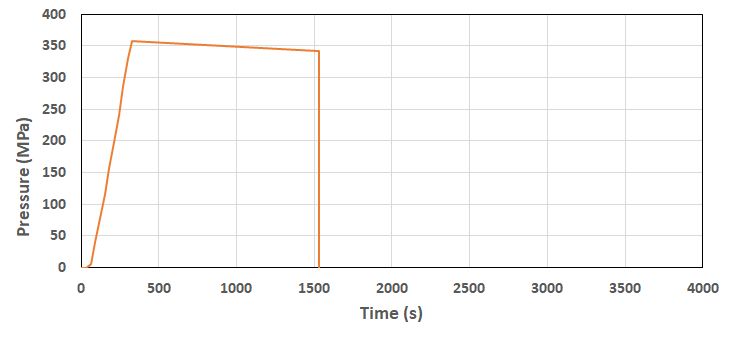

Supplement: Supplementary file 1 [file foods-12-04245-s001.zip › Figure S14_UCT_Experiment IV_Continuous pressurization.jpg]

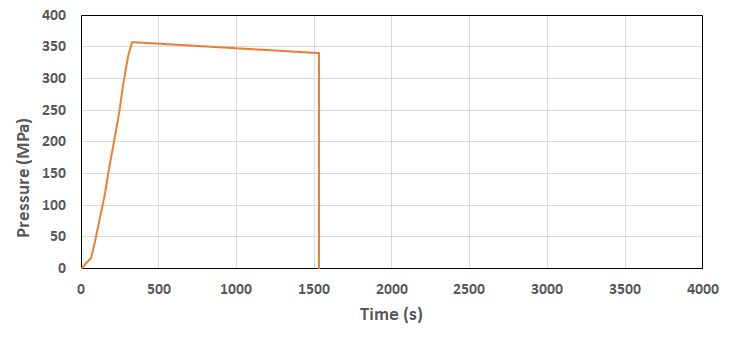

Supplement: Supplementary file 1 [file foods-12-04245-s001.zip › Figure S15_UCT_Experiment V_Continuous pressurization.jpg]

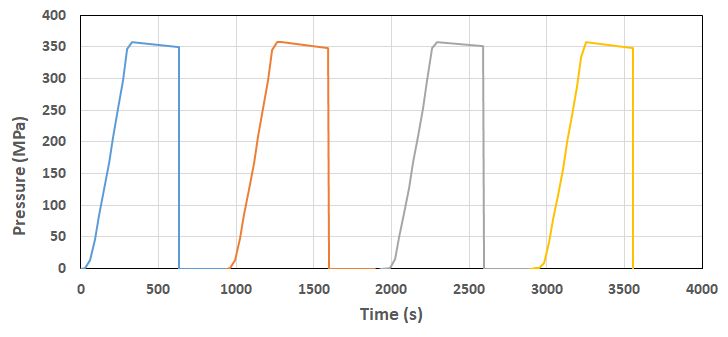

Supplement: Supplementary file 1 [file foods-12-04245-s001.zip › Figure S16_UCT_Experiment V_Chronological course of sample pressurization in 4 cycles with successive sampling.jpg]

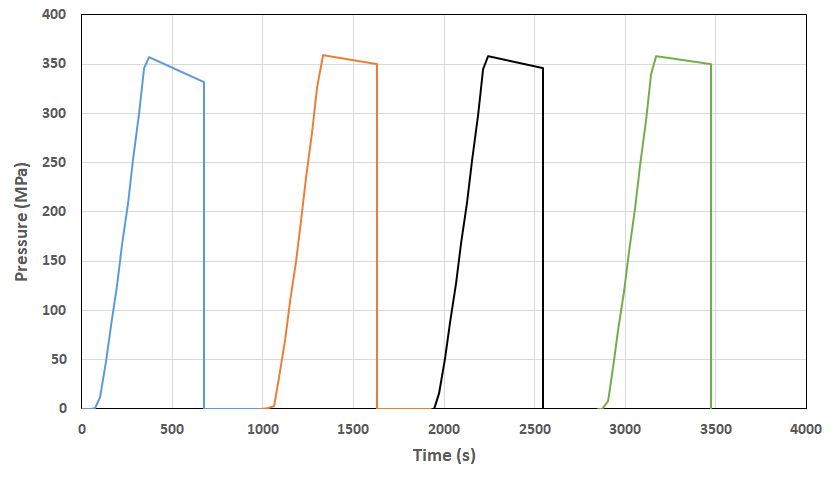

Supplement: Supplementary file 1 [file foods-12-04245-s001.zip › Figure S1_UH HK_Experiment VI_Pressurization in cycles.jpg]

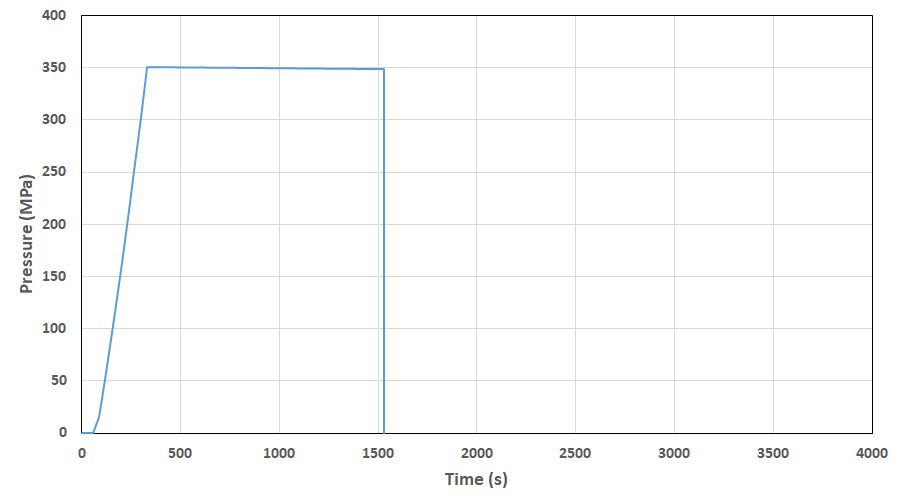

Supplement: Supplementary file 1 [file foods-12-04245-s001.zip › Figure S2_UHHK_Experiment VI_Continuous pressurization.jpg]

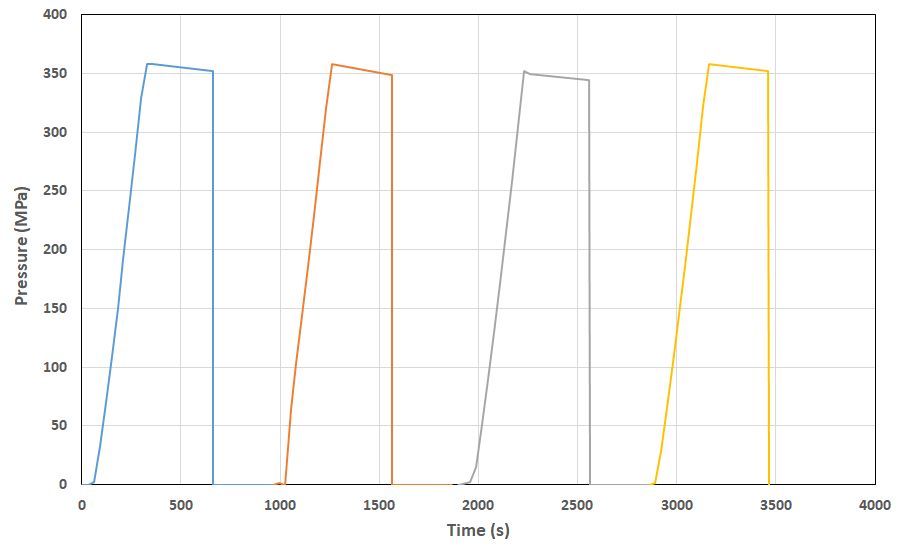

Supplement: Supplementary file 1 [file foods-12-04245-s001.zip › Figure S3_UHHK_Experiment VII_Pressurization in cycles.jpg]

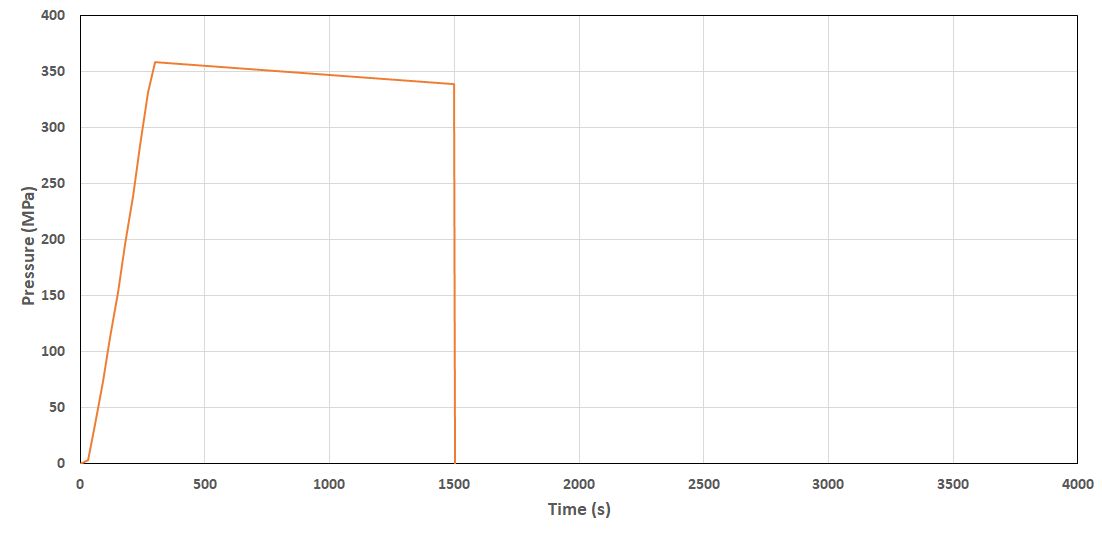

Supplement: Supplementary file 1 [file foods-12-04245-s001.zip › Figure S4_UH HK_Experiment VII_Continuous pressurization.jpg]

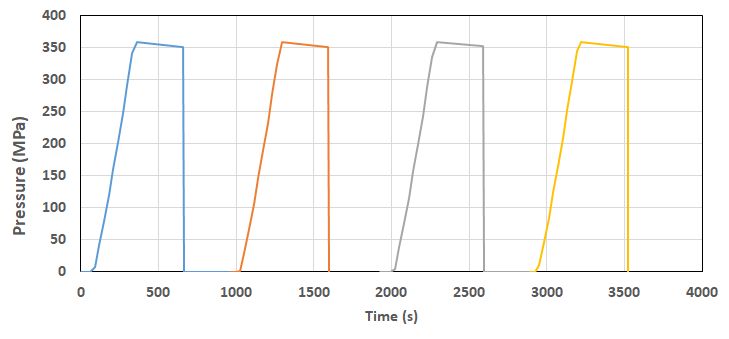

Supplement: Supplementary file 1 [file foods-12-04245-s001.zip › Figure S5_UH HK_Experiment VIII_Pressurization in cycles.jpg]

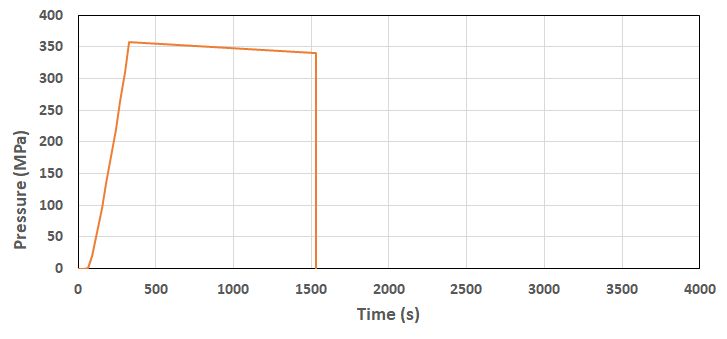

Supplement: Supplementary file 1 [file foods-12-04245-s001.zip › Figure S6_UH HK_Experiment VIII_Continuous pressurization.jpg]

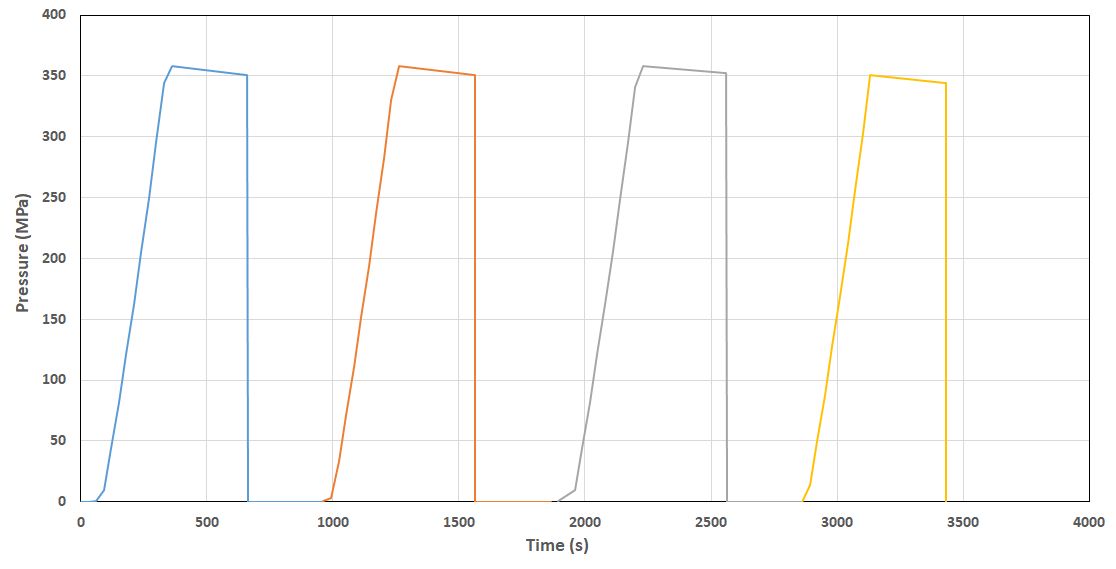

Supplement: Supplementary file 1 [file foods-12-04245-s001.zip › Figure S7_UCT_Experiment I_Pressurization in cycles.jpg]

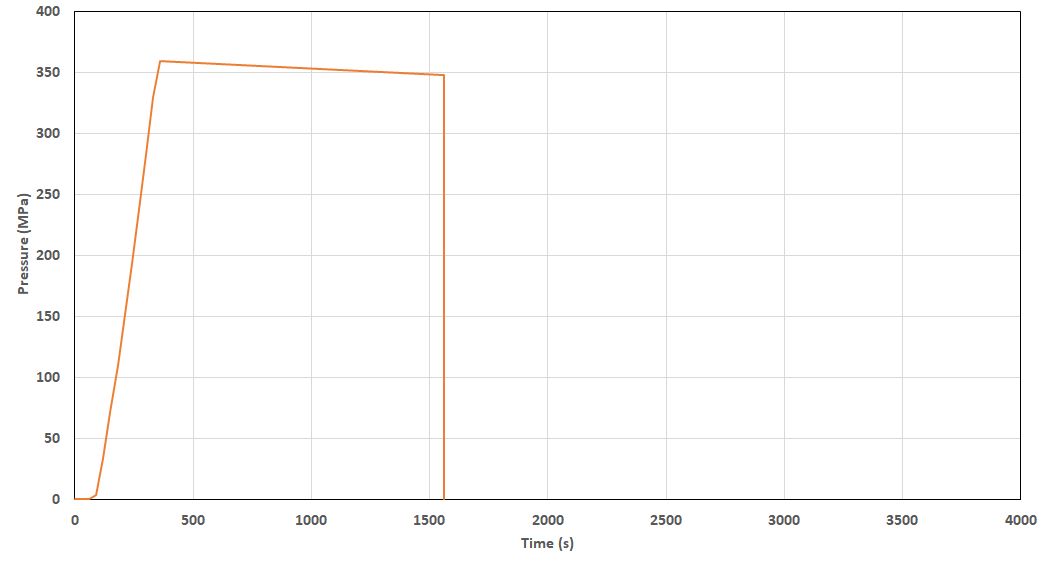

Supplement: Supplementary file 1 [file foods-12-04245-s001.zip › Figure S8_UCT_Experiment I_Continuous pressurization.jpg]

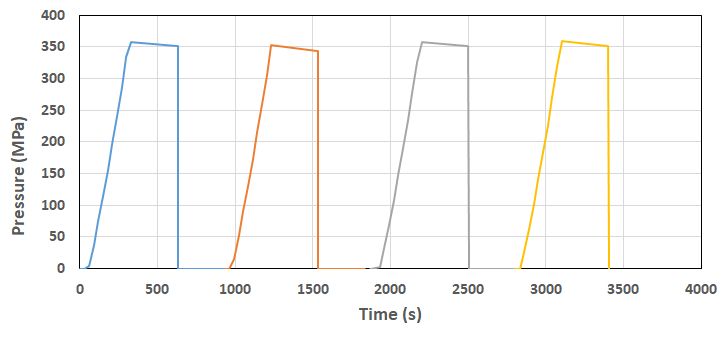

Supplement: Supplementary file 1 [file foods-12-04245-s001.zip › Figure S9_UCT_Experiment II_Pressurization in cycles.jpg]
